# Plasma Beta-Amyloid and Metabolic Biomarkers: Age- and Sex-Stratified Analyses Suggest Limited Population-Level Associations

**DOI:** 10.3390/medicina62050897

**Published:** 2026-05-06

**Authors:** Hyeokjun Yun, Bo Kyeung Jung, Jae Kyung Kim

**Affiliations:** 1 Department of Medical Laser, Graduate School of Medicine, Dankook University, Cheonan 31116, Republic of Korea; 10621yhj@naver.com; 2 Department of Laboratory Medicine, College of Medicine, Dankook University, Cheonan 31116, Republic of Korea; lovegodmother@hanmail.net; 3 Department of Biomedical Laboratory Science, College of Health Sciences, Dankook University, Cheonan 31116, Republic of Korea

**Keywords:** plasma beta-amyloid, metabolic biomarkers, inflammation, thyroid function, lipid metabolism, assay-based categorization, age- and sex-specific analysis

## Abstract

*Background and Objectives:* This study examined the relationships between plasma beta-amyloid levels and systemic inflammatory, thyroid, and lipid biomarkers using complementary analytical approaches in a cross-sectional cohort undergoing plasma OAβ and laboratory testing. *Materials and Methods:* Plasma beta-amyloid, high-sensitivity C-reactive protein (hsCRP), thyroid hormones (TSH and FT4), and lipid profiles (HDL, LDL, and TG) were analyzed. Pearson correlation analyses assessed continuous associations, and one-way analysis of variance (ANOVA) compared biomarker distributions across plasma beta-amyloid categories (low, boundary, high), with additional stratification by age and sex. Multivariable linear regression models adjusted for age and sex evaluated the independence of observed associations. *Results:* Overall, plasma beta-amyloid showed limited associations with metabolic biomarkers at the population level. A weak inverse correlation with hsCRP was observed in unadjusted analyses in the low group and in males, whereas other biomarkers showed no consistent associations. Additional correlations emerged in selected subgroups but were heterogeneous, based on small samples, and interpreted as exploratory. ANOVA-based comparisons showed no significant differences across plasma beta-amyloid categories in the overall cohort or major strata. Although HDL showed subgroup-specific variation, these associations were not retained after adjustment. *Conclusions:* Assay-based plasma beta-amyloid categorization was not associated with uniform systemic metabolic alterations. Common inflammatory, thyroid, and lipid biomarkers were not robust population-level correlates and, when present in subgroups, likely reflected context-dependent rather than stable associations.

## 1. Introduction

Alzheimer’s disease (AD), the leading cause of dementia worldwide, is neuropathologically characterized by extracellular deposition of beta-amyloid (Aβ) plaques and intracellular accumulation of hyperphosphorylated tau tangles [1,2]. In vivo quantification of cerebral amyloid pathology has traditionally relied on cerebrospinal fluid (CSF) biomarkers and amyloid positron emission tomography (PET), which are considered reference standards for assessing amyloid burden [1,3]. However, these approaches are limited by procedural invasiveness, high cost, and restricted feasibility in large-scale or longitudinal population-based studies [3]. In response to these limitations, blood-based biomarkers have emerged as attractive alternatives. Among them, plasma Aβ—particularly the Aβ42/Aβ40 ratio—has been increasingly investigated as a minimally invasive indicator of cerebral amyloid pathology and amyloid-related pathophysiological changes across the AD continuum [4,5,6].

Increasing evidence indicates that systemic metabolic and endocrine dysregulation may modulate amyloid metabolism and influence Alzheimer’s disease (AD) risk [7]. Chronic low-grade inflammation has been implicated in AD pathogenesis, with elevated circulating markers such as high-sensitivity C-reactive protein (hsCRP) associated with cognitive decline and disease progression in several cohorts [8]. However, associations between peripheral inflammatory markers and plasma Aβ levels remain inconsistent, with studies reporting positive, negative, or null relationships across different populations and study designs, underscoring the complexity of linking systemic inflammation to amyloid biology [9,10].

Thyroid dysfunction has been implicated as a potential modifier of amyloid metabolism and neurodegeneration in AD. Experimental evidence indicates that altered thyroid hormone signaling can change amyloid precursor protein processing and increase Aβ production in animal models [11]. Epidemiological and imaging studies have reported relationships between thyroid-stimulating hormone (TSH), free thyroxine (FT4), and dementia risk or cerebral amyloid burden, although results vary across cohorts [12,13]. Despite these observations, population-based evidence is inconsistent, and it remains unclear whether variation in peripheral thyroid hormone levels is reflected in circulating plasma Aβ when accounting for demographic factors such as age and sex.

Lipid metabolism is increasingly recognized as a pathway of interest in amyloid research. Several midlife and imaging-based cohort studies have linked dyslipidemia—particularly elevated low-density lipoprotein (LDL) cholesterol and triglycerides (TG)—to greater cerebral amyloid deposition or progression of AD pathology [14,15]. In contrast, other large population studies have found weak or null relationships between circulating lipid levels and amyloid burden measured by PET, underscoring heterogeneity in findings [16]. High-density lipoprotein (HDL) cholesterol, often considered protective in cardiovascular contexts, has shown mixed associations with amyloid outcomes and cognitive measures across studies, suggesting context-dependent effects influenced by age, sex, and disease stage [15,17].

Age and sex are well-established modifiers of both metabolic regulation and AD risk. Aging is associated with substantial alterations in inflammatory tone, endocrine signaling, and lipid metabolism, all of which may influence amyloid-related processes [18,19]. In addition, sex-specific differences in hormonal milieu and metabolic control have been shown to modulate susceptibility to AD pathology and clinical progression [20]. Nevertheless, many prior studies have evaluated metabolic–amyloid associations in pooled populations, which may mask age- or sex-dependent relationships.

Therefore, the present study aimed to investigate the associations between plasma beta-amyloid levels and systemic inflammatory, thyroid, and lipid metabolic biomarkers across predefined assay-based plasma beta-amyloid categories. To address potential heterogeneity, we additionally performed stratified analyses by age and sex using both correlation-based approaches and group comparisons. By integrating these complementary analytical frameworks, we sought to determine whether plasma beta-amyloid categorization was accompanied by discernible alterations in metabolic biomarkers and to identify demographic contexts in which such associations might emerge.

## 2. Materials and Methods

### 2.1. Study Design and Participants

**Figure 1:**
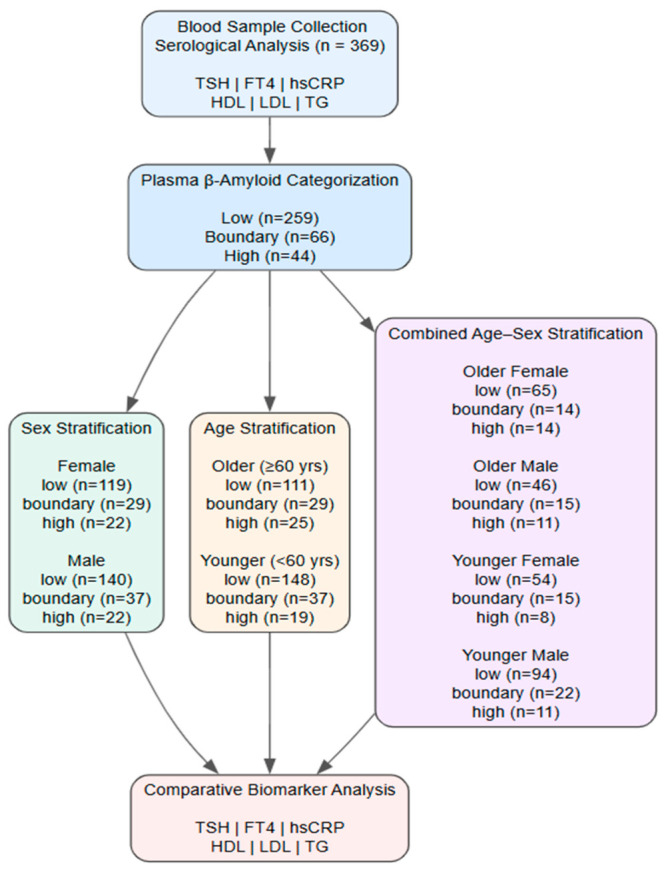
Flow diagram of participant selection and stratification. A total of 369 individuals were included in the final analysis and categorized according to age (<60 vs. ≥60 years), sex, and plasma beta-amyloid category groups (low, boundary, high).

A total of 369 participants were included in the final analysis, comprising 199 males and 170 females. Based on age classification, 204 participants were aged <60 years, and 165 were aged ≥60 years. According to plasma beta-amyloid categorization, 259 participants were classified as low, 66 as boundary, and 44 as high. Detailed stratification according to sex, age, and combined age–sex subgroups is presented in Figure 1. This cross-sectional study investigated associations between plasma beta-amyloid category and metabolic and inflammatory biomarkers. Following blood sample collection, serological analyses were performed to measure hsCRP, thyroid-related markers (TSH and FT4), and lipid profiles (HDL, LDL, and TG). Participants were categorized into low-, boundary-, and high-category plasma beta-amyloid groups based on assay-derived plasma beta-amyloid values. Comparative analyses were subsequently conducted across stratification schemes including sex (female and male), age (<60 years and ≥60 years), and combined age–sex subgroups (older female, older male, younger female, and younger male). Within each stratum, biomarker levels were compared across assay-based plasma beta-amyloid categories to evaluate whether metabolic and endocrine profiles differed according to plasma beta-amyloid grouping. This hierarchical stratification approach enabled the assessment of potential age- and sex-dependent heterogeneity in beta-amyloid-related metabolic profiles [19,21].

### 2.2. Plasma Oligomeric Beta-Amyloid Measurement and Assay-Based Categorization

Alzheimer’s disease-related plasma beta-amyloid status was assessed using the inBlood™ OAβ test (PeopleBio Inc., Seongnam, Republic of Korea), which quantifies plasma oligomeric beta-amyloid using a modified sandwich enzyme-linked immunosorbent assay (Multimer Detection System, MDS). In this method, plasma samples are incubated with synthetic Aβ to promote oligomer formation, and oligomeric beta-amyloid is selectively captured and detected by epitope-overlapping antibodies specific for the N-terminus of Aβ. Following incubation and wash steps, horseradish peroxidase-conjugated detection antibodies generate a chemiluminescent signal proportional to oligomerized Aβ levels. Luminescence signals were quantified using manufacturer-provided standard curves. For stratified analyses, participants were categorized according to manufacturer-recommended interpretive cutoffs based on a prior blinded validation study performed using the same assay platform [22]. Accordingly, plasma oligomeric beta-amyloid values were classified as low (<0.78 ng/mL), boundary (0.78–0.92 ng/mL), and high (≥0.93 ng/mL). However, in the present study, these groups were used as assay-based plasma beta-amyloid categories for analytical stratification rather than as pathologically confirmed Alzheimer’s disease states. Because categorization depends on assay-specific interpretive thresholds and manufacturer-provided standard curves, classification-related heterogeneity may arise, particularly for values near the cut points. In addition, the present study did not independently validate these plasma categories against PET imaging or CSF biomarkers. Therefore, these categories should be interpreted cautiously as platform-specific analytical groupings rather than definitive indicators of cerebral amyloid pathology.

### 2.3. hsCRP, TSH, and FT4 Measurements

Serum TSH and FT4 were measured using electrochemiluminescence immunoassays on the Elecsys^®^ e801 analyzer (Roche Diagnostics, Mannheim, Germany). TSH was assessed by a sandwich immunoassay and FT4 by a competitive assay, with analytical ranges of 0.005–100 µIU/mL and 0.023–7.77 ng/dL and reference intervals of 0.27–4.20 µIU/mL and 0.93–1.70 ng/dL, respectively [23].

hsCRP was measured using a latex-enhanced immunoturbidimetric assay on the cobas^®^ c702 analyzer (Roche Diagnostics, Mannheim, Germany), with an upper measuring limit of 350 mg/L. Low-level concentrations were detectable within the analytical sensitivity of the assay. All measurements were performed in a certified laboratory under standardized quality control procedures [24].

### 2.4. HDL Cholesterol, LDL Cholesterol, and TG Measurements

Serum concentrations of HDL cholesterol, LDL cholesterol, and TG were determined using enzymatic colorimetric techniques with the cobas^®^ c702 analyzer (Roche Diagnostics, Mannheim, Germany) in accordance with the manufacturer’s instructions. HDL cholesterol and LDL cholesterol were measured using homogeneous and direct enzymatic assays, respectively. All lipid concentrations are expressed in mg/dL and were analyzed as continuous variables. Measurements were performed under standardized quality control procedures [25].

### 2.5. Statistical Analysis

Pearson correlation analyses were initially used to assess linear associations between continuous variables on the original measurement scale for descriptive interpretability. Continuous variables are presented as mean ± standard deviation. Normality was assessed using visual inspection of histograms and the Shapiro–Wilk test. Because hsCRP and TG showed right-skewed distributions, robustness was additionally assessed using log-transformed values and nonparametric Sensitivity analyses using log-transformed hsCRP and TG values and Spearman correlation analyses showed broadly similar directional patterns and did not materially alter the interpretation of the findings; therefore, the primary results are presented using Pearson correlation coefficients for interpretability (Supplementary Table S2). Group comparisons across plasma beta-amyloid categories were performed using one-way analysis of variance (ANOVA). Given the unequal cell sizes and small counts in certain strata (notably the high-category group), these comparisons were treated as exploratory. Residual distributions were visually inspected for approximate symmetry; however, these results should be interpreted cautiously, as subgroup sparsity may reduce the stability of *p*-values. When overall ANOVA was nominally significant, Bonferroni-adjusted post hoc pairwise comparisons were performed. Multivariable linear regression analyses adjusted for age and sex were used to evaluate whether observed associations persisted after demographic adjustment. Each biomarker was entered into the regression model individually rather than jointly to avoid multicollinearity and to assess independent associations with plasma beta-amyloid. To account for multiple comparisons inherent in extensive subgrouping, *p*-values were adjusted using the Benjamini–Hochberg False Discovery Rate (FDR) procedure. Both nominal *p*-values and FDR-adjusted *p*-values are reported (Supplementary Table S2) to provide a transparent evaluation of potential false-positive risks. Accordingly, statistically significant findings in stratified analyses were interpreted conservatively as nominal exploratory signals, with greater emphasis placed on effect-size magnitude, direction, and consistency across analytic approaches rather than on nominal *p*-values alone. All statistical analyses were conducted using GraphPad Prism (version 7.00.159; Dotmatics, Boston, MA, USA) and Jamovi (version 2.6; The jamovi project, Sydney, Australia). Statistical significance was defined as a two-sided *p*-value < 0.05.

## 3. Results

### 3.1. Association Between Plasma Beta-Amyloid and Metabolic Biomarkers

Associations between plasma beta-amyloid and metabolic biomarkers were first examined using unadjusted correlation analyses in the overall cohort and in strata defined by plasma beta-amyloid category, age, and sex (Table 1). Across the overall analytical framework, most associations were weak, inconsistent, or absent. A modest inverse correlation between plasma beta-amyloid and hsCRP was observed in the low-category group and in males; however, this pattern was not reproduced consistently across other strata or biomarkers.

Additional nominally significant correlations emerged in some age- and sex-defined subgroups (Table 2), but these findings were heterogeneous in direction and restricted to relatively small strata. Given the extensive subgrouping and the limited size of several subgroups, particularly within the high-category group, these results should be interpreted as exploratory and hypothesis-generating. Moreover, because multiple subgroup correlations were examined across extensive subgroup strata, and none of the nominal associations remained significant after formal multiplicity adjustment using the FDR procedure, the possibility that some findings reflect sampling variability or false-positive results cannot be excluded.

After adjustment for age and sex in multivariable linear regression models, none of the inflammatory, thyroid, or lipid biomarkers showed an independent association with plasma beta-amyloid. Taken together, these results indicate that nominal subgroup-level signals were not robustly retained across analytic approaches and should therefore be viewed as hypothesis-generating rather than confirmatory.

Age- and sex-stratified analyses identified several nominal subgroup-specific correlations between plasma beta-amyloid and selected metabolic biomarkers; however, these associations were heterogeneous in direction, confined to small strata, and not supported consistently across analytic approaches (Table 2). Given the extensive subgrouping, limited subgroup sample sizes, and the fact that none of the nominal associations survived formal multiplicity adjustment using the FDR procedure (Supplementary Table S2), these findings should be interpreted as exploratory and hypothesis-generating rather than as evidence of stable subgroup-specific biological associations. No biomarker showed a statistically significant independent association after adjustment for age and sex with plasma beta-amyloid. Sensitivity analyses using log-transformed hsCRP and TG values in both correlation and regression analyses yielded comparable association patterns, indicating that the observed findings were not materially influenced by distributional skewness.

### 3.2. Association Between Plasma Beta-Amyloid Categories and Metabolic Biomarkers

**Figure 2:**
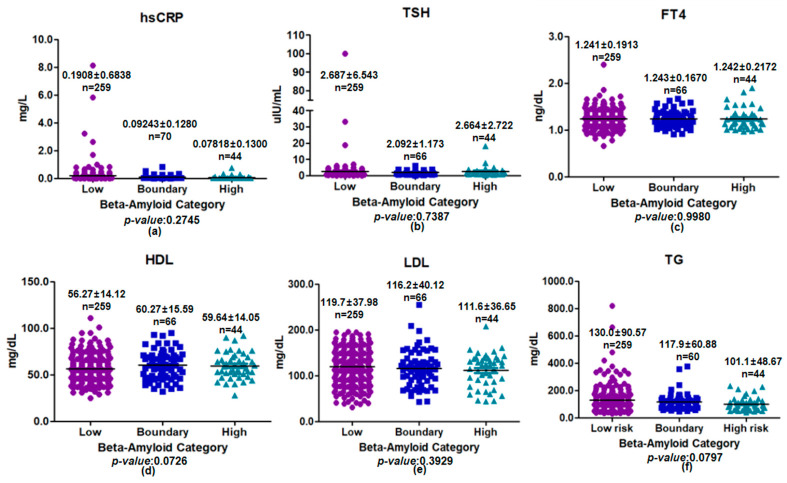
Distribution of plasma metabolic biomarkers according to plasma beta-amyloid categories (low, boundary, and high). Panels show levels of (**a**) hsCRP, (**b**) TSH, (**c**) FT4, (**d**) HDL, (**e**) LDL, and (**f**) TG. Data are presented as individual values with mean ± standard deviation. Group comparisons were performed using one-way analysis of variance. No statistically significant differences were observed among plasma beta-amyloid category groups (*p* > 0.05 for all comparisons).

Plasma metabolic biomarkers did not differ significantly across assay-based plasma beta-amyloid categories in the overall cohort (Figure 2). This absence of consistent group-level separation applied to inflammatory, thyroid, and lipid biomarkers, supporting the interpretation that plasma beta-amyloid categorization was not accompanied by a uniform systemic metabolic profile in this dataset. Thyroid-related markers (TSH and FT4) were comparable across plasma beta-amyloid categories (*p* = 0.7387 and *p* = 0.9980, respectively). Lipid biomarkers, including HDL, LDL, and TG, also showed no statistically significant differences across plasma beta-amyloid categories in the overall cohort, although modest variability was observed (HDL: *p* = 0.0726; LDL: *p* = 0.3929; TG: *p* = 0.0797). Overall, assay-based plasma beta-amyloid categorization was not accompanied by consistent group-level differences in inflammatory, thyroid, or lipid metabolic biomarkers in the overall cohort.

### 3.3. Association Between Plasma Beta-Amyloid Categories and Metabolic Biomarkers by Sex

**Figure 3:**
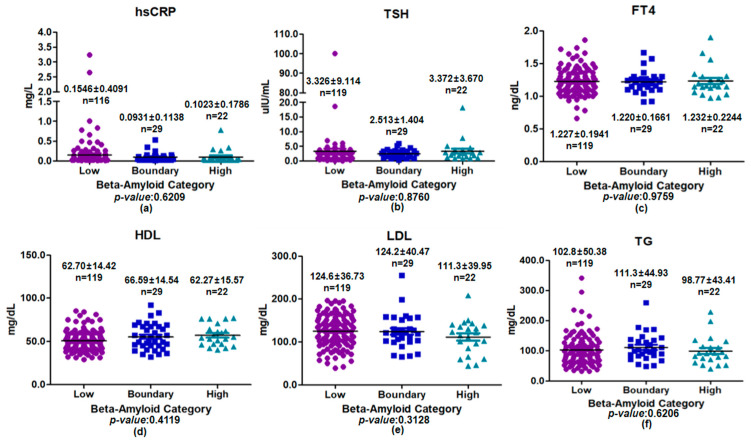
Comparison of inflammatory, thyroid, and lipid biomarkers across plasma beta-amyloid categories among female participants. Scatter plots with mean ± standard deviation are shown for (**a**) hsCRP, (**b**) TSH, (**c**) FT4, (**d**) HDL, (**e**) LDL, and (**f**) TG. Differences among low-, boundary-, and high groups were assessed using one-way analysis of variance. No statistically significant differences were observed for any biomarker (*p* > 0.05).

Among female participants, metabolic and inflammatory biomarkers were compared across plasma beta-amyloid categories (low, boundary, and high) (Figure 3). No statistically significant differences were observed for any biomarker across groups. Serum hsCRP showed a nonsignificant decreasing trend from the low group (0.1546 ± 0.4091 mg/L, *n* = 116) to the boundary (0.0931 ± 0.1138 mg/L, *n* = 29) and high groups (0.1023 ± 0.1786 mg/L, *n* = 22; *p* = 0.6209). TSH and FT4 levels were comparable across plasma beta-amyloid categories (*p* = 0.8760 and *p* = 0.9759, respectively). Lipid profiles also showed no significant differences, with similar HDL levels (*p* = 0.4119) and no group differences in LDL cholesterol or TG (*p* = 0.3128 and *p* = 0.6206, respectively). Overall, no consistent biomarker pattern was observed across plasma beta-amyloid categories in this subgroup, and any isolated nominal differences should be interpreted cautiously given the exploratory nature of the analysis.

**Figure 4:**
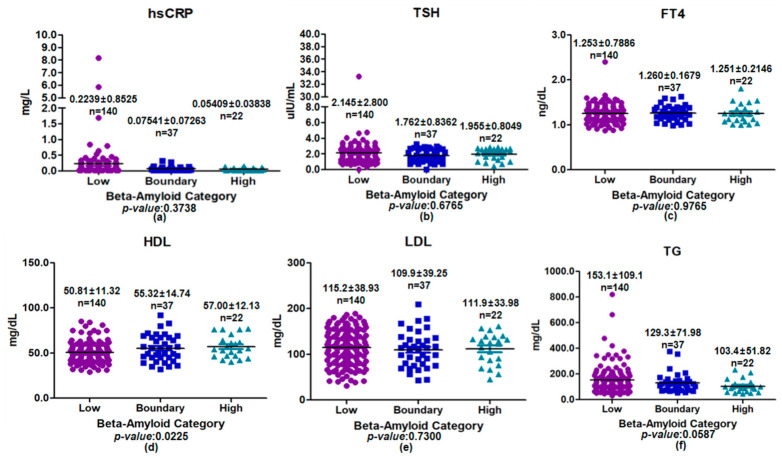
Comparison of inflammatory, thyroid, and lipid biomarkers across plasma beta-amyloid categories among male participants. Scatter plots with mean ± standard deviation are shown for (**a**) hsCRP, (**b**) TSH, (**c**) FT4, (**d**) HDL, (**e**) LDL, and (**f**) TG. Differences among low-, boundary-, and high groups were assessed using one-way analysis of variance. A significant difference was observed only for HDL cholesterol (*p* < 0.05), whereas all other biomarkers showed no statistically significant differences (*p* > 0.05).

Among male participants, metabolic and inflammatory biomarkers were compared across plasma beta-amyloid categories (low, boundary, and high) (Figure 4). Most biomarkers showed no clear differences according to plasma beta-amyloid category. Serum hsCRP exhibited a modest decreasing trend across plasma beta-amyloid categories, although this did not reach statistical significance (*p* = 0.3738). TSH and FT4 levels were broadly comparable across categories (*p* = 0.6765 and *p* = 0.9765, respectively), and LDL cholesterol likewise showed no apparent differences by plasma beta-amyloid categories (*p* = 0.7300). In male participants, HDL cholesterol showed a nominal overall group difference in the unadjusted ANOVA; however, no pairwise difference remained significant in post hoc testing, and the finding was not retained after demographic adjustment. Accordingly, this result should be interpreted as a non-robust exploratory signal rather than as evidence of an independent association. TG levels showed a decreasing trend with increasing plasma beta-amyloid category level, although this pattern did not reach conventional levels of statistical significance (*p* = 0.0587). Overall, no consistent biomarker pattern was observed across plasma beta-amyloid categories in this subgroup, and any isolated nominal differences should be interpreted cautiously given the exploratory nature of the analysis.

### 3.4. Association Between Plasma Beta-Amyloid Categories and Metabolic Biomarkers by Age

**Figure 5:**
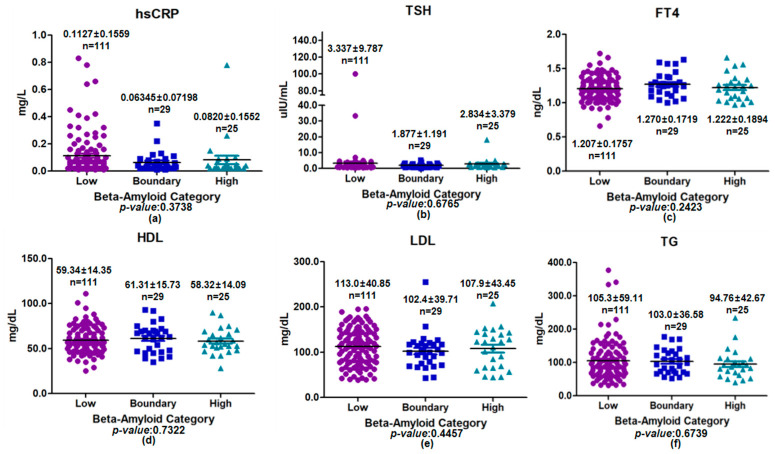
Comparison of inflammatory, thyroid, and lipid biomarkers across plasma beta-amyloid categories in the older group (≥60 years). Scatter plots with mean ± SD are shown for (**a**) hsCRP, (**b**) TSH, (**c**) FT4, (**d**) HDL, (**e**) LDL, and (**f**) TG. Differences among low-, boundary-, and high groups were assessed using one-way ANOVA. No statistically significant differences were observed for any biomarker (all *p* > 0.05).

In the older group (≥60 years), levels of hsCRP, TSH, FT4, HDL, LDL, and TG were analyzed according to plasma beta-amyloid category (low, boundary, and high) (Figure 5). In participants aged ≥60 years, none of the inflammatory, thyroid, or lipid biomarkers differed across plasma beta-amyloid categories (all *p* > 0.05). Although numerical variations were noted across plasma beta-amyloid categories, these differences did not reach statistical significance. Similarly, lipid-related parameters, including HDL, LDL, and TG, showed no significant associations with plasma beta-amyloid category (HDL: *p* = 0.7322; LDL: *p* = 0.4457; TG: *p* = 0.6739). Overall, inflammatory, thyroid-related, and lipid biomarkers remained comparable across plasma beta-amyloid categories in individuals aged ≥60 years.

**Figure 6:**
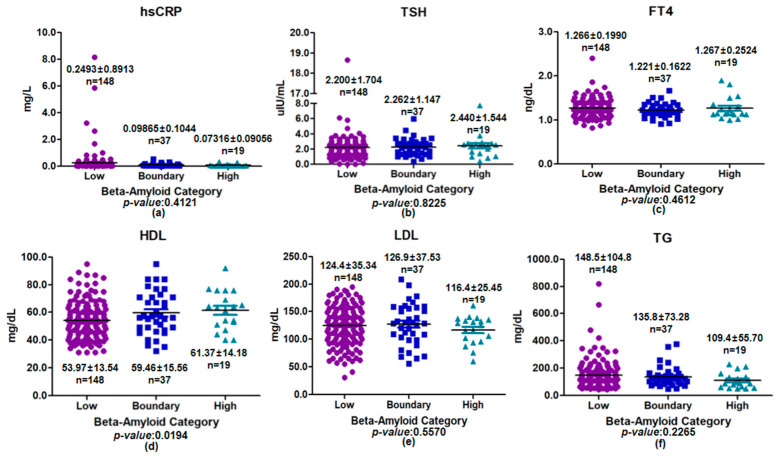
Comparison of inflammatory, thyroid, and lipid biomarkers across plasma beta-amyloid categories in the younger group (<60 years). Scatter plots with mean ± SD are shown for (**a**) hsCRP, (**b**) TSH, (**c**) FT4, (**d**) HDL, (**e**) LDL, and (**f**) TG. Differences among low-, boundary-, and high groups were assessed using one-way ANOVA. A significant difference was observed for HDL (*p* < 0.05), whereas all other biomarkers showed no statistically significant differences (*p* > 0.05).

In the younger group (<60 years), inflammatory, thyroid, and lipid biomarkers were compared across assay-based plasma beta-amyloid categories (low, boundary, and high) (Figure 6). Levels of hsCRP, TSH, and FT4 showed no clear differences among the three groups (hsCRP: *p* = 0.4121; TSH: *p* = 0.8225; FT4: *p* = 0.4612). Among lipid-related parameters, HDL cholesterol showed an overall difference across plasma beta-amyloid categories in the unadjusted analysis (ANOVA *p* = 0.0194). However, post hoc Bonferroni comparisons did not identify statistically significant pairwise differences between individual category groups. In contrast, LDL cholesterol and TG levels did not differ appreciably across plasma beta-amyloid categories (LDL: *p* = 0.5570; TG: *p* = 0.2265). Overall, no consistent biomarker pattern was observed across plasma beta-amyloid categories in this subgroup, and any isolated nominal differences should be interpreted cautiously given the exploratory nature of the analysis.

### 3.5. Association Between Plasma Beta-Amyloid Categories and Metabolic Biomarkers by Age and Sex

Age- and sex-stratified analyses were conducted to assess whether plasma beta-amyloid categories were associated with differential patterns of inflammatory, thyroid, and lipid biomarkers across demographic subgroups (Supplementary Figures S1–S4).

In older female participants (≥60 years; Supplementary Figure S1), none of the evaluated biomarkers—including hsCRP, TSH, FT4, HDL, LDL, and TG—differed significantly across plasma beta-amyloid categories (low, boundary, and high). Similarly, in older male participants (≥60 years; Supplementary Figure S2), no statistically significant differences were observed for any biomarker, although hsCRP and FT4 showed borderline numerical variation that did not reach statistical significance.

In younger female participants (<60 years; Supplementary Figure S3), all inflammatory, thyroid, and lipid biomarkers remained comparable across beta-amyloid categories, with no evidence of association. In younger male participants (<60 years; Supplementary Figure S4), most biomarkers likewise showed no significant differences; however, HDL cholesterol demonstrated a statistically significant difference across groups in unadjusted ANOVA, with higher levels observed in the high group compared with the low group in post hoc analysis. No other lipid or endocrine markers showed significant variation in this subgroup.

Overall, no consistent or reproducible biomarker pattern was identified across plasma beta-amyloid categories in any age–sex subgroup. Observed variations were limited, heterogeneous, and not consistently supported across analytic approaches. These findings indicate that assay-based plasma beta-amyloid categorization was not associated with stable subgroup-specific alterations in systemic inflammatory, thyroid, or lipid biomarkers in this dataset. Given the exploratory nature of subgroup analyses and the potential for small-sample effects, isolated nominal differences—such as the HDL finding in younger males—should be interpreted cautiously.

## 4. Discussion

In this cross-sectional cohort of individuals undergoing plasma OAβ and laboratory biomarker testing, we evaluated the relationships between plasma oligomeric beta-amyloid and systemic inflammatory, thyroid, and lipid biomarkers using a layered analytical framework. This included correlation analyses, group comparisons, adjusted regression models, and stratification by age and sex. The principal finding was that plasma beta-amyloid showed limited and non-uniform associations with these commonly measured biomarkers at the population level. Across the overall cohort, most correlations were weak or absent, ANOVA-based comparisons across plasma beta-amyloid categories showed little evidence of consistent group separation, and none of the tested biomarkers retained an independent association with plasma beta-amyloid after adjustment for age and sex. Together, these results indicate that broad and uniform systemic metabolic alteration was not a prominent correlate of plasma beta-amyloid levels in this cohort.

The principal contribution of this study lies not in identifying a single novel biomarker association, but in showing that subgroup-level associations are often not retained at the broader population level or after demographic adjustment. This conclusion is supported by a combined stratified and method-comparative analytical framework. In this sense, the value of the study is methodological as well as descriptive: it highlights how stratified exploratory findings can differ from broader population-level patterns and underscores the need for caution when interpreting nominal subgroup associations in heterogeneous biomarker research. In unadjusted correlation analyses, plasma beta-amyloid showed a weak inverse association with hsCRP in the low group and among males, while most other biomarkers were not significantly correlated with beta-amyloid. When analyses were further stratified by age, sex, and beta-amyloid category, additional significant correlations emerged in selected subgroups, including associations involving LDL cholesterol, FT4, TSH, and triglycerides. However, these findings were heterogeneous in direction and confined to relatively small subgroups. Because extensive subgroup stratification increases susceptibility to sampling variability and multiplicity-related false-positive findings, these results are best interpreted as exploratory signals rather than robust biological patterns. This interpretation is further supported by the regression analyses, in which none of the biomarkers showed independent associations with plasma beta-amyloid after adjustment for age and sex.

An important caution concerns the extensive subgroup analyses performed in this study. Although stratification by age and sex was biologically motivated, several subgroup cells were small, particularly within the high plasma beta-amyloid category. Under these conditions, statistically significant findings may be unstable and vulnerable to random variation. In addition, because multiple subgroup correlations were examined across multiple strata and none remained significant after formal multiplicity adjustment using the FDR procedure, the nominally significant findings in Table 2 may reflect sampling variability and multiplicity-related false-positive results. Accordingly, these analyses should be interpreted as exploratory and not as confirmatory evidence of subgroup-specific biological associations. These findings are therefore best viewed as hypothesis-generating observations that require validation in larger, prespecified datasets.

The discrepancy between isolated subgroup correlations and the largely null population-level ANOVA findings provides additional insight. Correlation analyses assess continuous covariation within strata, whereas ANOVA tests whether predefined plasma beta-amyloid category groups differ sufficiently in mean biomarker levels to produce meaningful group-level separation. In the present study, most subgroup-level associations were not strong or consistent enough to translate into clear differences across plasma beta-amyloid categories. This pattern suggests that even when metabolic biomarkers show statistically detectable relationships with plasma beta-amyloid in selected strata, those relationships are likely modest and context-dependent rather than representing stable determinants of plasma beta-amyloid variation.

Inflammatory markers demonstrated particularly limited associations with plasma beta-amyloid. Although hsCRP exhibited inverse correlations with beta-amyloid in certain contexts—specifically within the low group and among younger males—these associations were not consistently observed across other strata and did not persist after demographic adjustment. This pattern is consistent with previous reports describing heterogeneous and context-dependent relationships between systemic inflammation and amyloid-related pathology, with studies variably reporting positive, null, or inverse associations between inflammatory biomarkers and amyloid burden [26,27,28,29]. Taken together, these findings suggest that systemic inflammatory markers measured in peripheral blood may have only limited relevance for explaining variation in circulating beta-amyloid levels in community-based populations.

Thyroid-related biomarkers similarly demonstrated inconsistent correlation patterns. Opposite-direction associations between beta-amyloid and TSH or FT4 were observed in selected male subgroups depending on age and beta-amyloid category, but these findings lacked consistency across sex and age strata. Previous studies have reported associations between thyroid hormone levels and amyloid or tau pathology [11,12,30,31]. However, the variability observed in the present study supports the interpretation that peripheral thyroid hormones may exert age- or sex-dependent modulatory effects rather than serving as stable systemic biomarkers of amyloid burden. This interpretation is further supported by Mendelian randomization analyses suggesting a potential protective role of genetically predicted TSH levels, whereas evidence for FT4 remains inconclusive [32].

Lipid metabolism showed similarly heterogeneous patterns. LDL cholesterol and triglycerides did not demonstrate consistent relationships with plasma beta-amyloid across the study population. HDL cholesterol, however, showed subgroup-specific associations in selected analyses, particularly among younger males. These findings should be interpreted cautiously. No consistent HDL association was observed in the overall cohort, the direction of the associations varied across strata, and the signals were not retained after adjustment for age and sex. Accordingly, these HDL-related findings are more plausibly explained by demographic structure, residual confounding, or stochastic variation arising from stratified analyses rather than reflecting a stable independent biological relationship.

Nevertheless, previous studies provide biologically plausible mechanisms linking HDL metabolism to amyloid biology. HDL and its major apolipoprotein component, apoA-I, have been implicated in amyloid-β binding, peripheral transport, and clearance, suggesting a potential role in modulating circulating beta-amyloid levels. Prior studies have reported divergent associations between lipid markers and amyloid pathology depending on biological compartment, disease stage, and demographic characteristics [33,34,35]. Observational analyses have also reported that extremely high HDL concentrations may be associated with increased dementia risk in large population cohorts, although genetic evidence for a causal relationship remains limited [35,36]. Conversely, epidemiological and mechanistic studies have suggested protective effects of HDL or apoA-I on Alzheimer’s disease risk, possibly through vasoprotective mechanisms and facilitation of amyloid clearance [36]. Longitudinal evidence further suggests that both low and very high HDL levels may be associated with increased dementia risk compared with intermediate levels, implying non-linear and context-dependent relationships influenced by age, sex, and HDL functionality [33,34,35,36].

The age- and sex-stratified analyses provide an additional exploratory perspective. Peripheral biomarker behavior often differs according to demographic context, and the present findings suggest that plasma beta-amyloid–metabolic relationships may be conditional rather than universal. Interestingly, the absence of significant associations among individuals aged ≥60 years across all biomarkers may indicate that peripheral metabolic signatures become attenuated as neurodegenerative processes progress or as systemic aging effects accumulate. In contrast, the presence of HDL-related signals in younger males raises the possibility that metabolic–amyloid interactions may be more detectable earlier in the disease continuum, before extensive central pathology develops. However, the current data do not support the inference that any demographic subgroup possesses a reproducibly distinct metabolic signature of plasma beta-amyloid. Replication in larger cohorts with prespecified subgroup hypotheses will be required to determine whether such demographic modulation truly exists [37,38].

From an interpretive perspective, the largely null associations observed in this study indicate that clear independent relationships between assay-based plasma beta-amyloid categorization and common inflammatory, thyroid, or lipid biomarkers were not detectable under the present study conditions. These findings should not be interpreted to mean that such systemic factors are biologically irrelevant to amyloid-related processes; rather, they suggest that broad and uniform population-level associations were not evident in this dataset. Given the cross-sectional design, assay-specific categorization, limited subgroup sizes, and residual confounding, the present results do not permit conclusions regarding biomarker robustness, diagnostic utility, or the relative specificity of plasma beta-amyloid for central amyloid pathology [38].

Several limitations should be considered when interpreting the present findings. First, although the overall sample size was moderate, the number of participants in the high beta-amyloid category was relatively small (*n* = 44), and additional stratification by age and sex substantially reduced the sample size in several subgroups. In such small strata, correlation coefficients may be unstable and sensitive to outliers, potentially yielding inflated effect size estimates [39,40].

Second, this study employed a cross-sectional design, which precludes any inference regarding causal relationships [41,42]. The observed associations between plasma beta-amyloid and metabolic or endocrine biomarkers may reflect downstream consequences, shared regulatory mechanisms, or parallel processes rather than direct causal effects [43]. Longitudinal studies are therefore needed to clarify temporal dynamics and directionality.

Third, another important limitation concerns the interpretation of plasma oligomeric beta-amyloid itself. The inBlood™ OAβ assay provides a peripheral blood-based measurement generated within a specific assay platform and does not constitute direct evidence of PET- or CSF-confirmed cerebral amyloidosis. In addition, categorization based on manufacturer-recommended cut-offs may introduce classification variability, particularly near threshold boundaries. Measurement heterogeneity may also arise from assay calibration and standard curve-based quantification. Although the categorization strategy used in this study was based on a prior blinded validation study employing the same platform, the present cohort did not undergo independent validation against central amyloid biomarkers. Prior studies of the inBlood™ OAβ platform have demonstrated acceptable analytical performance and concordance with established amyloid biomarkers, including reported associations with amyloid PET positivity and clinical diagnostic classification. These validation data support the utility of the assay as a peripheral screening or risk-stratification tool, although it does not replace direct measures of cerebral amyloid pathology. Therefore, the plasma beta-amyloid groups used here should be interpreted as assay-based analytical categories rather than definitive Alzheimer’s disease risk states or pathological classifications. This distinction is important to avoid overgeneralization from peripheral assay findings to established Alzheimer’s disease pathology [43,44,45].

Fourth, metabolic and hormonal biomarkers were assessed at a single time point. Given the well-documented intra-individual variability of hsCRP, thyroid hormones, and lipid parameters, single measurements may not fully capture long-term inflammatory or metabolic status [46,47,48]. Such measurement variability can result in regression dilution, potentially attenuating true associations between biomarkers and plasma beta-amyloid levels [49].

Fifth, although several key covariates were considered in the analyses, residual confounding cannot be excluded. In particular, medication use for hypertension and diabetes was not systematically controlled. Antihypertensive agents, glucose-lowering medications, and related metabolic therapies may influence lipid profiles, inflammatory markers, and possibly amyloid-related pathways. The absence of detailed pharmacologic adjustment represents a significant limitation and may have contributed to subgroup-specific variability. Addressing this limitation prospectively would strengthen future investigations. In addition, medication use such as lipid-lowering therapy or thyroid hormone replacement was not comprehensively captured. These treatments are known to substantially affect circulating lipid concentrations and metabolic markers, potentially modifying observed associations. Lifestyle factors—including diet, smoking status, alcohol consumption, and physical activity—were also not fully incorporated into the analytical models. Because these behaviors are closely linked to both cardiometabolic risk and systemic inflammation, residual confounding remains possible. Importantly, apolipoprotein E (APOE) genotype was not available in the present cohort. APOE polymorphism is a well-established genetic determinant of amyloid biology and Alzheimer’s disease risk. However, the objective of this study was to evaluate baseline correlations between plasma oligomeric beta-amyloid and metabolic biomarkers in a general community screening context, where genetic testing is not routinely performed. Therefore, the findings should be interpreted as providing real-world population-level associations rather than genotype-stratified mechanistic insights [12,50,51]. In addition, parameters related to glucose metabolism, including fasting glucose, insulin levels, and measures of insulin resistance, were not available in the present dataset. This represents an important limitation, as insulin resistance has been mechanistically and epidemiologically linked to amyloid-β accumulation and Alzheimer’s disease pathology. Experimental and clinical studies have demonstrated that impaired insulin signaling may promote amyloidogenesis, reduce amyloid-β clearance via insulin-degrading enzyme, and contribute to neuroinflammation and oxidative stress pathways relevant to amyloid pathology [52,53]. Furthermore, population-based studies have reported that higher insulin resistance is associated with increased cerebral amyloid deposition even in cognitively normal individuals [54]. Therefore, future studies incorporating comprehensive metabolic profiling, including glucose homeostasis and insulin resistance indices, will be important to more fully characterize the relationship between metabolic dysfunction and plasma beta-amyloid.

Sixth, the observed associations—particularly those involving HDL cholesterol—were restricted to specific age- and sex-defined subgroups and were not consistently replicated across broader population analyses, indicating context-dependent patterns in lipid–amyloid and cognitive relationships. Prior cohort and cross-sectional studies have demonstrated heterogeneity in HDL associations with dementia risk or cognitive outcomes, with some showing positive links in certain age/sex strata while others report null or conflicting results across the full sample [55,56,57]. These findings highlight substantial biological variability and limited generalizability of subgroup-specific associations in metabolic–amyloid relationships.

Seventh, this study has additional limitations related to potential residual confounding from systemic and pharmacologic factors that were not fully captured. Lipid-lowering therapies and anti-inflammatory agents can significantly alter both lipid profiles and circulating inflammatory markers; for example, anti-inflammatory treatment with canakinumab has been shown to reduce levels of hsCRP [58]. Adiposity and elevated body mass index have been independently associated with higher hsCRP levels and dyslipidemia [59]. Insulin resistance and type 2 diabetes are closely linked to abnormalities in triglyceride and HDL cholesterol concentrations [60]. Thyroid dysfunction also exerts a significant influence on lipid metabolism, with hypothyroidism associated with increased LDL cholesterol and alterations in HDL and triglyceride concentrations [61]. Moreover, acute-phase reactants such as hsCRP are modulated by a range of systemic and therapeutic factors beyond underlying pathology [62]. The largely null findings should also be interpreted with caution. The absence of robust associations in this dataset does not necessarily indicate that inflammatory, thyroid, or lipid-related factors are irrelevant to plasma beta-amyloid biology. Rather, these results indicate that clear independent associations were not detectable under the present study conditions. Residual confounding remains likely because medication use, adiposity, smoking, alcohol intake, physical activity, and APOE genotype were not comprehensively available. These factors may have attenuated, obscured, or modified true associations and therefore limit the interpretability of both nominal subgroup signals and null overall results [51,63,64].

Despite these limitations, the present study has several important strengths. This study systematically evaluated the relationships between plasma beta-amyloid and inflammatory, thyroid, and lipid metabolic biomarkers using both correlation-based analyses and population-level group comparisons, with comprehensive stratification by beta-amyloid category, age, and sex. This dual analytical framework allowed us to distinguish subgroup-specific correlations from population-wide metabolic patterns, thereby reducing the risk of overinterpretation based on a single analytical approach. Importantly, the largely null findings observed in the one-way ANOVA analyses across plasma beta-amyloid categories support the interpretation that plasma beta-amyloid categorization was not accompanied by broad or uniform alterations in systemic metabolic or endocrine markers in this dataset, thereby contributing useful negative evidence to a field characterized by heterogeneous and sometimes contradictory results. Moreover, the presence of isolated nominal associations in selected demographic strata underscores the value of age- and sex-aware exploratory analyses. However, because these signals were not consistent across biomarkers or analytic approaches and arose from limited subgroup samples, they should be regarded as provisional observations rather than as evidence of reproducible demographic modulation of plasma beta-amyloid–metabolic relationships [20,35,37,38,43,65,66].

The principal contribution of this study lies not in identifying a single novel biomarker association but in showing that apparent subgroup-level signals were not consistently retained across correlation analyses, group comparisons, and adjusted models. In this respect, the study provides a method-comparative perspective on a heterogeneous literature and supports a more cautious interpretation of peripheral metabolic correlates of assay-based plasma beta-amyloid.

In conclusion, assay-based plasma beta-amyloid categorization was not accompanied by uniform changes in systemic inflammatory, thyroid, or lipid biomarkers in this cross-sectional cohort of individuals undergoing plasma OAβ and laboratory biomarker testing. Most observed associations were weak, heterogeneous, or confined to small demographic subgroups, and none remained independently associated with plasma beta-amyloid after adjustment for age and sex. These findings suggest that common metabolic biomarkers did not function as consistent population-level correlates of plasma beta-amyloid in this dataset and analytical framework. Because subgroup analyses were exploratory and important confounders were not fully captured, larger longitudinal studies with PET- or CSF-based validation, genetic information, and more detailed metabolic profiling are needed.

## Figures and Tables

**Table 1:** Correlation between plasma beta-amyloid and metabolic biomarkers across plasma beta-amyloid categories, age groups, and sex.

	Subgroup	*n*	Biomarker	Pearson r	95% CI	*p*-Value	Significance
Beta-amyloid categories	Low	259	hsCRP	−0.1834	−0.2995 to −0.0619	0.0033	Yes
			TSH	0.0214	−0.1018 to 0.1438	0.7344	No
			FT4	−0.0671	−0.1884 to 0.0563	0.2861	No
			HDL	0.074	−0.0493 to 0.1951	0.2388	No
			LDL	0.0506	−0.0727 to 0.1724	0.4211	No
			TG	0.0208	−0.1023 to 0.1433	0.7411	No
	Boundary	66	All biomarkers	−0.10–0.14	Includes 0	>0.26	No
	High	44	All biomarkers	−0.21–0.05	Includes 0	>0.16	No
Age	older (≥60 years)	165	All biomarkers	−0.06–0.13	Includes 0	>0.43	No
	Younger (<60 years)	204	All biomarkers	−0.13–0.04	Includes 0	>0.06	No
Sex	Male	199	hsCRP	−0.1614	−0.2940 to −0.0228	0.0227	Yes
			Other biomarkers	−0.09–0.12	Includes 0	>0.08	No
	Female	170	All biomarkers	−0.13–0.03	Includes 0	>0.66	No

**Table 2:** Exploratory age- and sex-stratified correlations between plasma beta-amyloid levels and metabolic or inflammatory biomarkers across assay-based plasma beta-amyloid categories. Pearson correlation coefficients (r) and two-tailed *p*-values are presented. All age-, sex-, and plasma beta-amyloid category strata are shown. Statistically significant nominal associations (*p* < 0.05) are reported where present; strata without nominally significant findings are indicated as NS. All analyses are exploratory. None of the observed associations remained statistically significant after false discovery rate (FDR) adjustment (see Supplementary Table S2).

Age	Sex	Plasma Beta-Amyloid Category Group	Sample Size	Significant Biomarker	Direction	r	*p*-Value	FDR-Adjusted *p*
Older	Female	Low	65	–	–	–	NS	NS
	Female	Boundary	14	–	–	–	NS	NS
	Female	High	14	LDL	Positive	0.6375	0.0142	0.0852
	Male	Low	46	TSH	Negative	−0.3022	0.0412	0.2472
	Male	Low	46	FT4	Positive	0.3162	0.0323	0.1938
	Male	Boundary	11	–	–	–	NS	NS
	Male	High	11	TG	Positive	0.7014	0.0162	0.0972
Younger	Female	Low	54	–	–	–	NS	NS
	Female	Boundary	15	–	–	–	NS	NS
	Female	High	8	LDL	Negative	−0.7973	0.0178	0.1068
	Male	Low	94	hsCRP	Negative	−0.2493	0.0154	0.0924
	Male	Boundary	22	FT4	Positive	0.5287	0.0114	0.0684
	Male	High	11	–	–	–	NS	NS

## Data Availability

The datasets utilized and examined in this study can be obtained from the corresponding author upon reasonable request.

## Supplementary Materials

The following supporting information can be downloaded at: https://www.mdpi.com/article/10.3390/medicina62050897/s1, Table S1: Multivariable linear regression analyses examining associations between plasma β-amyloid levels (dependent variable) and metabolic or inflammatory biomarkers.; Table S2: Summary of correlation analyses between plasma beta-amyloid and biomarkers across subgroups; Figure S1: Comparison of inflammatory, thyroid, and lipid biomarkers across plasma β-amyloid categories in older female participants (≥60 years); Figure S2: Comparison of inflammatory, thyroid, and lipid biomarkers across plasma β-amyloid categories in older male participants (≥60 years); Figure S3: Comparison of inflammatory, thyroid, and lipid biomarkers across plasma β-amyloid categories in younger female participants (<60 years); Figure S4: Comparison of inflammatory, thyroid, and lipid biomarkers across plasma β-amyloid categories in younger male participants (<60 years).
