# Supplementary material for: Plasma Beta-Amyloid and Metabolic Biomarkers: Age- and Sex-Stratified Analyses Suggest Limited Population-Level Associations
Source: Medicina (Kaunas). 2026 May 6;62(5):897. doi: 10.3390/medicina62050897 (PMC13208843; doi:10.3390/medicina62050897)
Supplement: Supplementary file 1 [file medicina-62-00897-s001.zip › medicina-4224676-supplementary.pdf]

Supplementary Table S1. Multivariable linear regression analyses examining associations between plasma β-amyloid levels (dependent variable) and metabolic or inflammatory biomarkers. Regression coefficients (β) and standard errors (SE) are presented. Each biomarker was entered into a separate regression model adjusted for age and sex. hsCRP and TG were log-transformed due to right-skewed distributions. Model R^2 values indicate the proportion of variance explained. Statistical significance was defined as a two-sided $p < 0.05$. Plasma β-amyloid was analyzed as a continuous variable (ng/mL); due to variability in assay-derived values, regression coefficients may appear numerically large relative to typical reference ranges.

**Model (Outcome: Plasma β-amyloid)**	**Predictor**	**β (Estimate)**	**SE**	**p-value**
**hsCRP (log-transformed)**	hsCRP	-0.381	1.838	0.836
	Age	0.003	0.092	0.97
	Sex	2.256	2.149	0.294
	**Model R^2**		**0.0033**	
**TSH**	TSH	-0.06	0.19	0.751
	Age	0.006	0.091	0.949
	Sex	2.332	2.159	0.281
	**Model R^2**		**0.0035**	
**FT4**	FT4	2.436	5.603	0.664
	Age	0.009	0.092	0.921
	Sex	2.317	2.154	0.282
	**Model R^2**		**0.0037**	
**HDL**	HDL	0.007	0.079	0.933
	Age	0.005	0.091	0.958
	Sex	2.193	2.311	0.343
	**Model R^2**		**0.0032**	
**LDL**	LDL	-0.047	0.028	0.099
	Age	-0.026	0.093	0.777
	Sex	2.808	2.166	0.196
	**Model R^2**		**0.0106**	
**TG (log-transformed)**	TG	-0.141	2.146	0.948
	Age	0.004	0.093	0.967
	Sex	2.348	2.199	0.31
	**Model R^2**		**0.0032**	

Supplementary Table S2. Summary of correlation analyses between plasma beta-amyloid and biomarkers across subgroups.

Age	Sex	Group	Significant Biomarker	Direction	r	Nominal p	FDR-adjusted p
Older	Female	Low	–	–	–	NS	NS
	Female	Boundary	–	–	–	NS	NS
	Female	High	LDL	Positive	0.6375	0.0142	0.0852
	Male	Low	TSH	Negative	-0.3022	0.0412	0.2472
	Male	Low	FT4	Positive	0.3162	0.0323	0.1938
	Male	Boundary	–	–	–	NS	NS
	Male	High	TG	Positive	0.7014	0.0162	0.0972
Younger	Female	Low	–	–	–	NS	NS
	Female	Boundary	–	–	–	NS	NS
	Female	High	LDL	Negative	-0.7973	0.0178	0.1068
	Male	Low	hsCRP	Negative	-0.2493	0.0154	0.0924
	Male	Boundary	FT4	Positive	0.5287	0.0114	0.0684
	Male	High	–	–	–	NS	NS

Note. FDR-adjusted p-values were calculated across all subgroup correlation tests using the Benjamini-Hochberg procedure. Nominal p-values < 0.05 are shown, but no variables reached the formal significance threshold of $P_{FDR} < 0.05$.

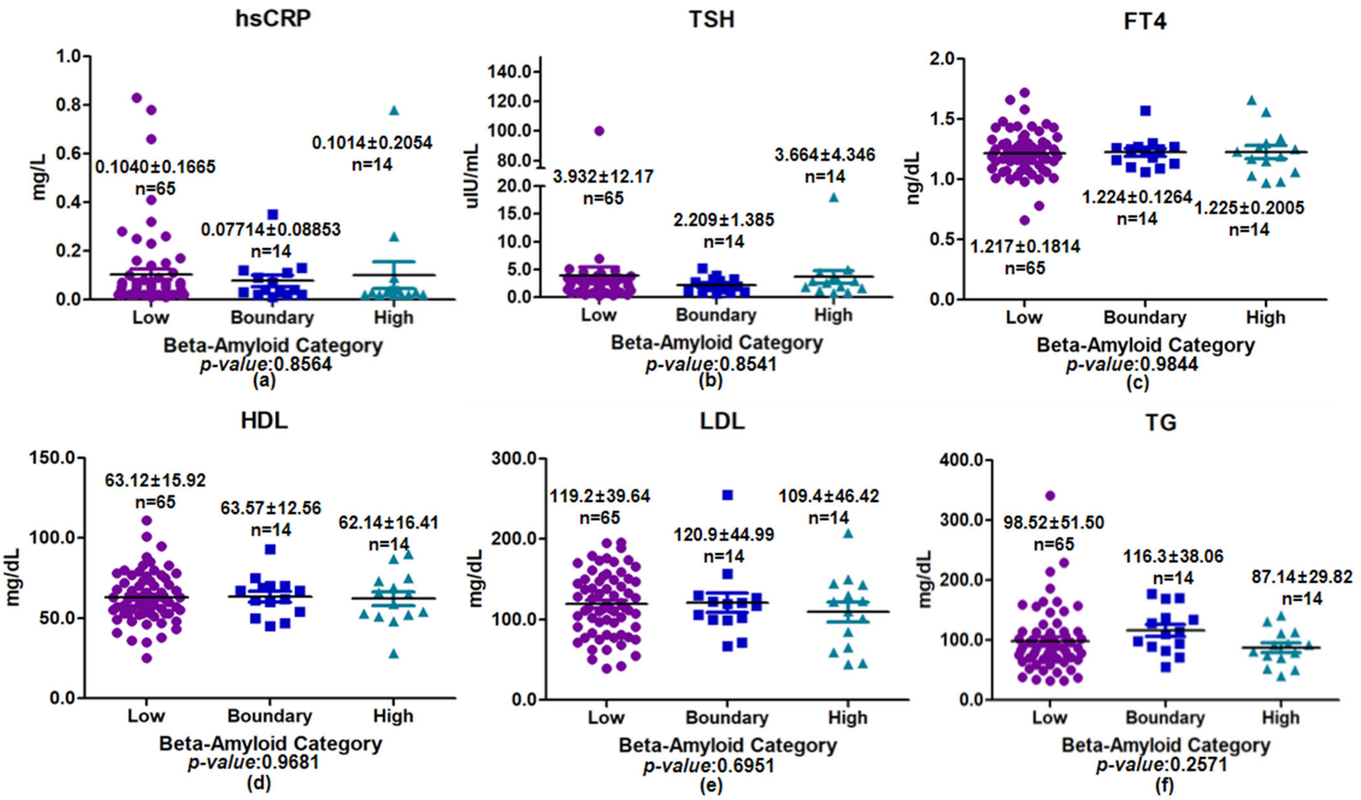

Supplementary Figure S1. Comparison of inflammatory, thyroid, and lipid biomarkers across plasma β-amyloid categories in older female participants (⩾60 years). Scatter plots with mean ± SD are shown for (a) hsCRP, (b) TSH, (c) FT4, (d) HDL, (e) LDL, and (f) TG. Differences among low, boundary, and high groups were assessed using one-way ANOVA. No statistically significant differences were observed for any biomarker (all $p > 0.05$).

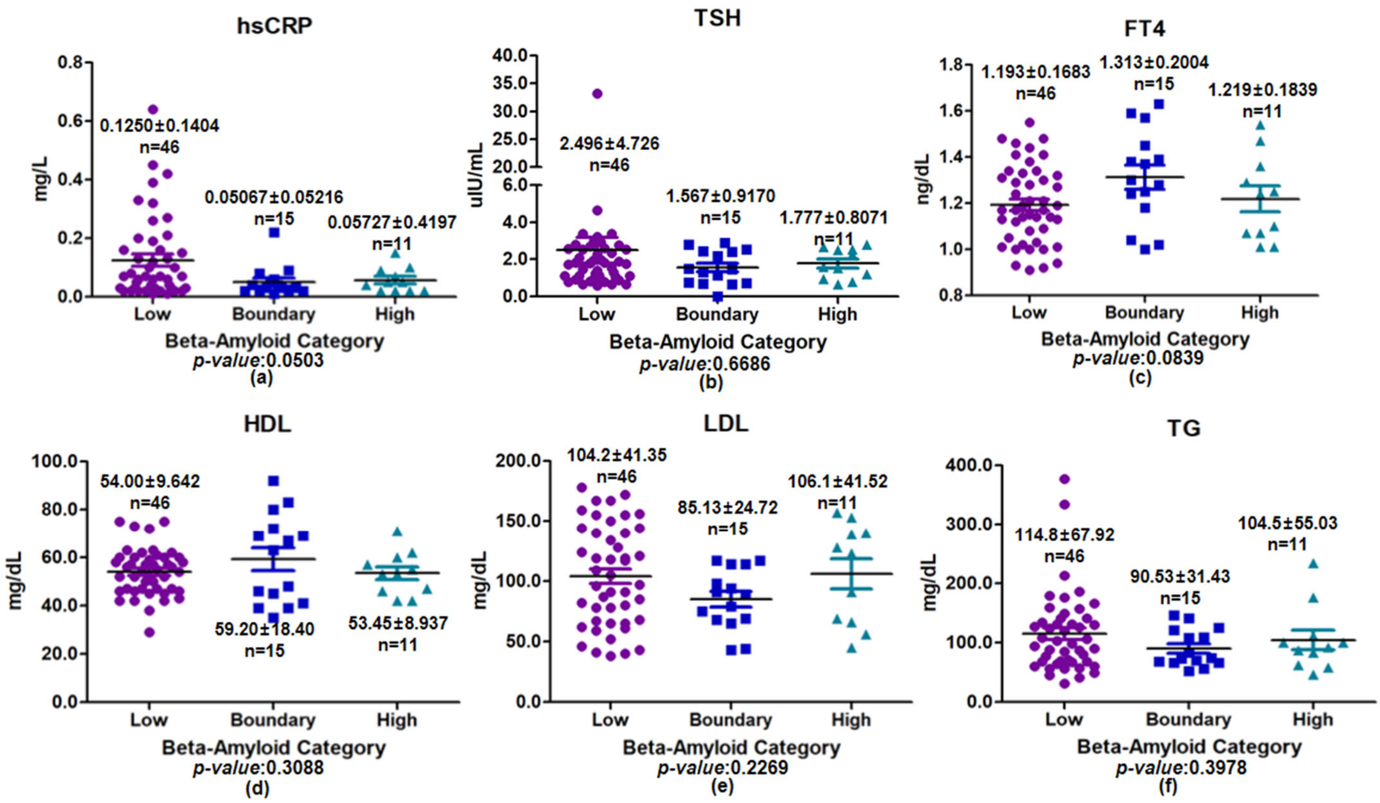

Supplementary Figure S2. Comparison of inflammatory, thyroid, and lipid biomarkers across plasma β-amyloid categories in older male participants (⩾60 years). Scatter plots with mean ± SD are shown for (a) hsCRP, (b) TSH, (c) FT4, (d) HDL, (e) LDL, and (f) TG. Differences among low, boundary, and high groups were assessed using one-way ANOVA. No statistically significant differences were observed for any biomarker (all $p > 0.05$).

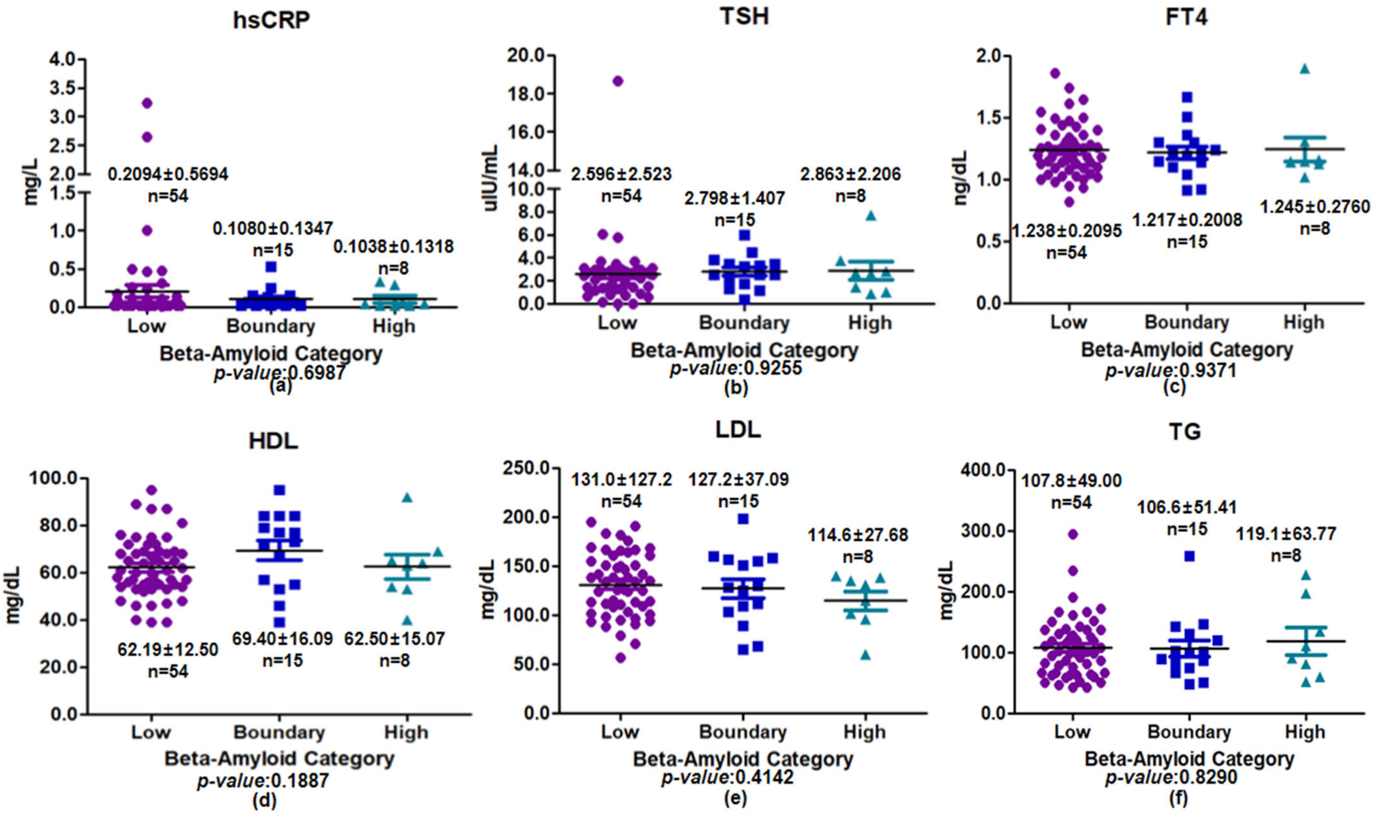

Supplementary Figure S3. Comparison of inflammatory, thyroid, and lipid biomarkers across plasma β-amyloid categories in younger female participants (<60 years). Scatter plots with mean ± SD are shown for (a) hsCRP, (b) TSH, (c) FT4, (d) HDL, (e) LDL, and (f) TG. Differences among low, boundary, and high groups were assessed using one-way ANOVA. No statistically significant differences were observed for any biomarker (all $p > 0.05$).

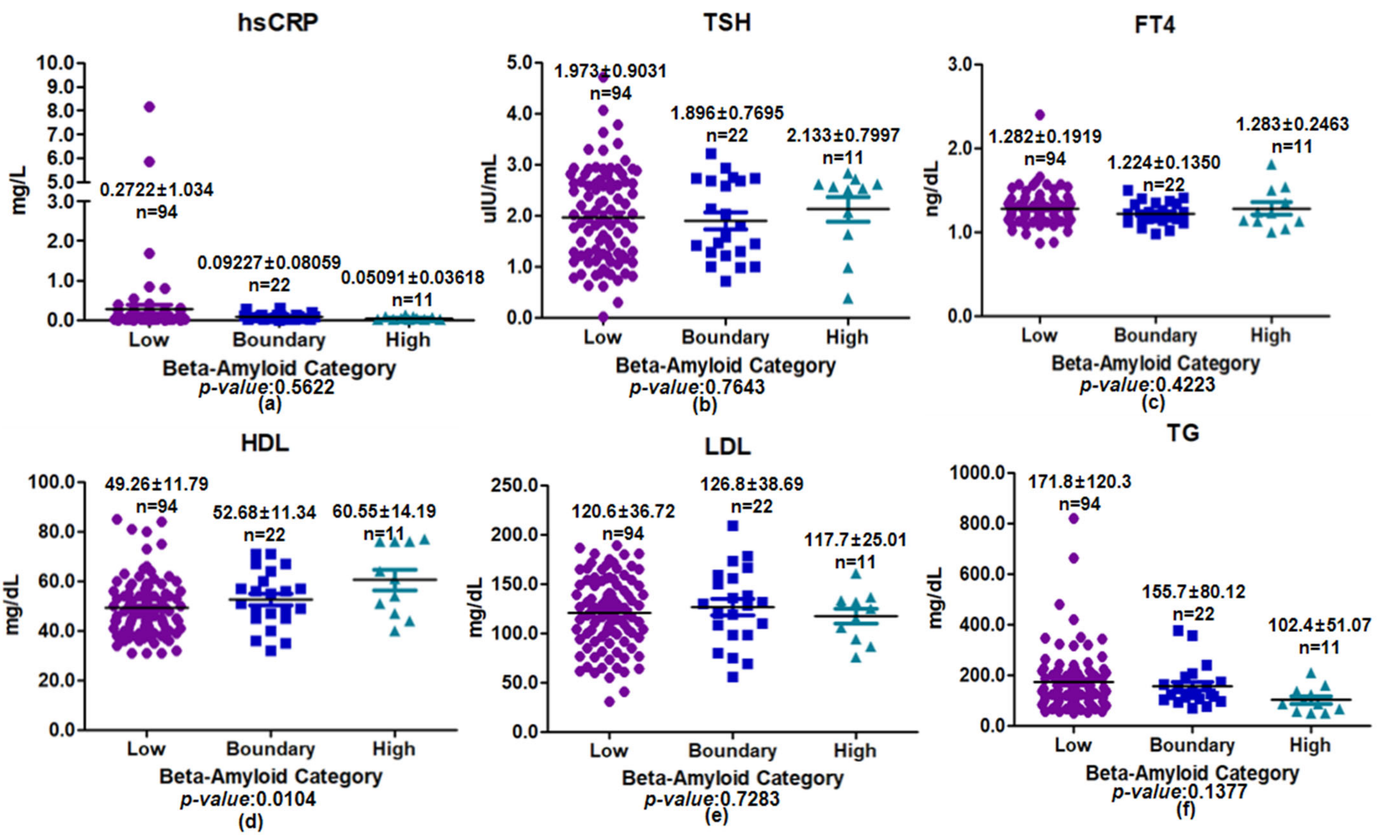

Supplementary Figure S4. Comparison of inflammatory, thyroid, and lipid biomarkers across plasma β-amyloid categories in younger male participants (<60 years). Scatter plots with mean ± SD are shown for (a) hsCRP, (b) TSH, (c) FT4, (d) HDL, (e) LDL, and (f) TG. Differences among low, boundary, and high groups were assessed using one-way ANOVA. A significant difference was observed only for HDL levels (p = 0.0104), while other biomarkers showed no statistically significant differences.
